# The incidence of incisional hernia after aortic aneurysm is not higher than after benign colorectal interventions: A retrospective control-matched cohort study

**DOI:** 10.1007/s00772-018-0390-z

**Published:** 2018-06-06

**Authors:** A. Wiegering, D. Liebetrau, S. Menzel, C. Bühler, R. Kellersmann, U. A. Dietz

**Affiliations:** 1 Klinik und Poliklinik für Allgemein‑, Viszeral‑, Gefäß- und Kinderchirurgie, Universitätsklinikum Würzburg, Würzburg, Germany; 2 Lehrstuhl für Biochemie und Molekularbiologie, Universität Würzburg, Würzburg, Germany; 3 Klinik für Viszeral‑, Gefäss- und Thoraxchirurgie, Kantonsspital Olten, Baselstr. 150, 4600 Olten, Switzerland

**Keywords:** Benign colon resection, Abdominal wall closure, Abdominal aortic aneurysm, Incisional hernia, Median laparotomy, Kolonresektion, Bauchdeckenverschluss, Aortenaneurysma, Narbenhernie, Mediane Laparotomie

## Abstract

**Background:**

Abdominal aortic aneurysms (AAA) have most probably an inflammatory origin, whereby the elastica is the layer actually involved. In the past, collagen weackness was supposed to be the shared cause of both, AAA and incisional hernias. Since the development of new techniques of closure of the abdominal wall over the last decade, collagen deficency seems to play only a secondary etiologic role.

**Objectives:**

The aim of the study was to investigate whether the incidence of incisional hernia following laparotomy due to AAA differs from that of colorectal interventions.

**Material and methods:**

This was a retrospective control matched cohort study. After screening of 403 patients with colorectal interventions and 96 patients with AAA, 27 and 72 patients, respectively were included. The match criteria for inclusion of patients with colorectal interventions were: age, benign underlying disease and median xiphopubic laparotomy. The primary endpoint was the incidence of an incisional hernia. The secondary endpoints were the risk profile, length of stay in the intensive care unit and postoperative complications. Data analysis was carried in the consecutive collective from 2006 to 2008.

**Results:**

In the group with AAA the mean follow-up was 34.5±18.1 months and in the group with colorectal interventions 35.7±21.4 months. The incidence of incisional hernias showed no significant differences between the two groups. In the AAA group 10 patients (13.8%) developed an incisional hernia in contrast to 7 patients in the colorectal intervention group (25.9%).

**Conclusions:**

In our collective patients with AAA did not show an increased incidence of incisional hernia in comparison to patients with colorectal interventions with comparable size of the laparotomy access and age. The quality of closure of the abdominal wall seems to be an important factor for the prevention of incisional hernia.

## Zusammenfassung

**Hintergrund:**

Das abdominelle Aortenaneurysma (AAA) hat eine inflammatorische Genese, wobei die Elastica die eigentlich beteiligte Schicht ist. In den vergangenen Jahrzehnten galt die Hypothese, dass die Kollagenqualität für beide, AAA und Narbenhernien verantwortlich sei. Mit der Entwicklung neuer Techniken des Bauchdeckenverschlusses im Laufe der letzten Jahrzehnte, scheint die Bedeutung des defizitären Kollagens als kausale Ursache abgenommen zu haben.

**Fragestellung:**

Die Studie geht der Frage nach, ob sich die Inzidenzrate von Narbenhernien nach Laparotomie aufgrund eines AAA oder kolorektalen Eingriffs unterscheidet.

**Material und Methoden:**

Dies ist eine retrospektive Matched-control-Kohortenstudie. Nach Screening von 403 kolorektalen Patienten und 96 AAA Patienten wurden 27 und 72 Patienten eingeschlossen. Match-Kriterien für den Einschluss kolorektaler Patienten waren: Alter, benigne Grunderkrankung und median-xiphopubische Laparotomie. Das primäre Ergebnis war die Inzidenz einer Narbenhernie im Verlauf. Sekundäre Ergebnisse waren das Risikoprofil, der Aufenthalt auf Intensivstation und postoperative Komplikationen. Die Datenanalyse erfolgte im konsekutiven Kollektiv der Jahre 2006 bis 2008.

**Ergebnisse:**

In der Gruppe der AAA betrug das mittlere Follow-up 34,5 ± 18,1 Monate, in der Gruppe der kolorektalen Eingriffe 35,7 ± 21,4 Monate. Die Narbenhernieninzidenz in beiden Gruppen unterschied sich nicht signifikant: In der AAA-Gruppe hatten 10 Patienten (13,8 %) eine Narbenhernie entwickelt, wohingegen 7 kolorektale Patienten (25,9 %) eine Narbenhernie ausbildeten.

**Schlussfolgerungen:**

Im eigenen Patientenkollektiv haben Patienten mit AAA im Vergleich zu kolorektalen Patienten mit vergleichbar großem Laparotomiezugang und Alter keine erhöhte Narbenhernieninzidenz. Die Qualität des Bauchdeckenverschlusses scheint ein wichtiger Faktor bei der Prävention der Narbenhernie zu sein.

## Introduction

The development of incisional hernia following abdominal surgery is an undesired and, depending on the findings, high-risk event. Its incidence ranges between 2% and 15% in the literature [1–3]. There are a number of theories on the etiology of incisional hernia, with the techniques of abdominal closure and collagen metabolism being the two most important. The latter is based on the assumption that the development of incisional hernia is triggered by a disproportion of type I collagen to type III [4, 5]. It was also long suspected that aortic aneurysms were caused by a collagen defect [6] and that these patients were at particularly high risk for developing incisional hernia; however, according to the current state of knowledge on the development of aortic aneurysms, collagen metabolism is no longer considered to be a major triggering factor. Thus, from a clinical perspective, the hypothesis can be postulated that the incidence of incisional hernia following medial xiphopubic laparotomy is not higher in abdominal aortic aneurysm patients than in matched controls with benign colorectal disease.

This hypothesis is based on the following rationales:

- Incisional hernias develop differently depending on the incision and need to be differentially classified and grouped. Medial xiphopubic laparotomy is performed for abdominal aortic surgery. Therefore, it is essential that this patient group be compared exclusively with a control group that has also undergone medial xiphopubic laparotomy; patients serving as the control group should have no recognizable endogenous risk factors for incisional hernia [7].
- From an historical perspective, it is assumed that the pathogenesis of abdominal aortic aneurysms (AAA) is based on impaired collagen production, which, at the same time as being an endogenous risk factor, also increases the risk of incisional hernias [6].
- The pathogenesis of AAA remains unclear. Novel experimental investigations show that it is probably not caused by a collagen defect as previously assumed [8] but arises instead from the Tunica intima.
- As a result, the historical assumption that the increased incidence of incisional hernia in aortic aneurysm patients is due to a collagen defect needs to be reviewed, and the incisional hernia incidence in this patient group can possibly be attributed to some other causality.

Collagen deficency is probably not the etiology of abdominal aortic aneurysms

In order to answer the working hypothesis, this article examines the following questions:

- Are there differences in the incidence of incisional hernia at 1 year (primary endpoint) between the two groups?
- What are the differences between the two groups in terms of the secondary endpoints: operating time, intensive care stay, postoperative complications, and inpatient stay?
- What perspectives result from answering the study question in terms of further clinical and experimental research?

## Patients and methods

The study cohort comprised all patients undergoing a surgical aortic aneurysm procedure or colorectal procedure at the Surgical Department of the University Hospital of Würzburg between 1 January 2006 and 31 December 2008. Due to the differing surgical indications, the patient cohort comprising 499 cases was divided into two groups for comparison, with 96 cases belonging to the aortic aneurysm group and 403 cases to the colorectal procedure group.

In order to ensure comparability of the two groups, the following inclusion criteria were used to achieve a valid matched control. The inclusion criteria were: age >57 and <77 years (MW ± SD of age in the AAA group), medial xiphopubic laparotomy (European Hernia Society [EHS] classification: M1 + M2 + M3 + M4 + M5), and benign resected tissue on histology [9].

A total of 96 cases were evaluated in the aortic aneurysm group. Of the 24 cases that were excluded, 17 had already died by the time the study was conducted and no post-operative follow-up had been documented. In a further two cases, no follow-up dates could be determined despite attempts to contact these patients both in writing and by telephone. The remaining five cases that were excluded consisted of: one case that was reported twice, one case declared to be an aortic aneurysm but which was treated with a colorectal procedure, one case in which an aortic aneurysm and the colon were operated on in a single-stage approach and two cases in which the indications for aortic aneurysm repair using stent grafts were established. This left a total of 72 complete records for evaluation.

**Fig. 1:**
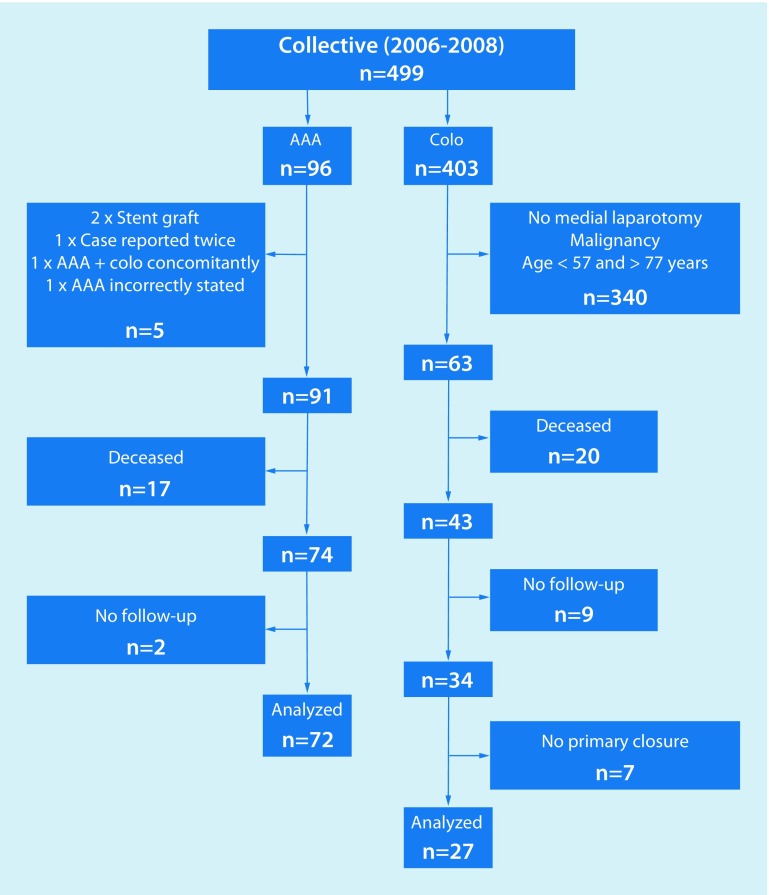
Patient flow diagram (*AAA* abdominal aortic aneurysm, *Colo* colorectal procedure)

The colorectal procedure group comprised 403 potential cases. Once the inclusion criteria and the abovementioned age matching (> 57 and < 77 years) had been applied, 63 cases remained. Of these 63 cases, 20 had already died by the time the study was conducted and no post-operative follow-up had been documented. In a further nine cases, no follow-up date could be determined despite attempts to contact these patients both in writing and by telephone. A further seven cases were excluded as they had not undergone primary abdominal wall closure. This left a total of 27 complete records for evaluation (Table 1; Fig. 1).

**Table 1:** Patient collective

Patient collective	Patients		Aortic aneurysm		Colorectal procedure	
	*n*	%	*n*	%	*n*	%
*Total*	499	100	96	–	403	–
*Patients included*	99	19.8	72	75	27	6.7
*Excluded*	400	80.2	24	25	367	93.3
Failed to meet inclusion criteria	345	86.3	5	20.8	340	90.4
Deceased	37	9.3	17	70.8	20	5.3
No primary fascial closure	7	1.8	–	–	7	1.9
Loss to follow-up	11	2.8	2	8.4	9	2.4

### Preoperative data

The surgical indications in the aortic aneurysm group were symptomatic aortic aneurysm or aneurysm measuring over 5 cm in diameter and showing annual progressive growth of over 0.5 cm in diameter. Patients in the colorectal group were selected on the basis of the appropriate surgical procedure, verification in the surgical report that a medial xiphopubic laparotomy had indeed been performed, and exclusion of oncological disease.

The risk factors for the development of incisional hernia were determined. Preoperative risk factors included: male gender, obesity with a body mass index (BMI) of over 30 kg/m^2^, nicotine use, diabetes mellitus, hypertension, kidney failure, cortisone use, lung disease, preoperative hemoglobin below 10 g/dl, preoperative albumin below 3.5 g/dl, a known pre-existing hernia, known cancer, collagen disorders, emergency surgical procedures, known previous surgery, as well as a second procedure within 1 month. Pulmonary diseases included chronic obstructive pulmonary disease (COPD), chronic bronchitis, and bronchial asthma. Postoperative risk factors were divided into two categories: particular features of the postoperative course and postoperative complications [2, 10].

### Medial laparotomy and abdominal closure in both patient groups

Laparotomy was performed using a skin incision around the left side of the umbilicus from below the xiphoid process to immediately above the pubic symphysis; incision of subcutaneous tissue and sparing exposure of the linea alba; dividing the linea alba with a scalpel and opening the peritoneum.

Abdominal wall closure was performed using a continuous absorbable monofilament 1-USP loop suture while maintaining a suture-to-wound length ratio of 4:1 [11]. At least two loops were used: starting in the suprapubic region, the first was used as a continuous suture to the navel, and the second, beginning in the subxiphoid region, also as a continuous suture to the umbilicus, where the two loops were knotted together. After rinsing the subcutis with saline solution, a subcutaneous redon drain was placed and the skin incision closed using polypropylene USP 3‑0 Donati stitches. The precise length or distance from the xiphoid process to the symphysis was not routinely measured.

### Primary outcome

The occurrence of incisional hernia was defined as the primary endpoint. The follow-up date was determined from the previous medical report and any available diagnostic computed tomography (CT) images were also evaluated in the diagnosis. The date of the CT scan was set as the follow-up date. If no postoperative course had been documented, patients were contacted in writing.

### Secondary outcomes

Postoperative complications were divided into two categories. Postoperative risk factors were assigned to an “intensive medicine” category and a “non-intensive medicine” category. The former included wound infections, catecholamine therapy, relaparotomy, artificial ventilation, and the length of stay on the intensive care unit. Non-intensive medical complications included infections, seroma, and secondary wound healing. These complications were classified according to the Clavien–Dindo classification [12].

### Follow-up examination

Follow-up examinations of patients after AAA repair were planned for 3, 6, and 12 months as standard at the Surgical Department of the University Hospital Würzburg. Following benign colorectal procedures, patients were examined at 1 year. Patients who did not attend follow-up at the University Hospital Würzburg were contacted in writing and asked to complete a questionnaire. Beyond the 12-month period, the latest clinical outcome of medial abdominal wall closure was noted.

### Statistics

Data were collected in an Excel® (Microsoft, Redmon, WA, USA) table and then coded. Statistical analysis was carried out using SPSS Statistic 24® (IBM, Armonk, NY, USA). In all, 99 records were analyzed in which follow-up could be determined. In the univariate analysis, the relevance of the individual factors was calculated in a statistical paired comparison using the exact Fisher test. This made it possible to determine whether two variables were independent of or dependent on each other. The relationship was considered significant at a bilateral significance level of *p* < 0.05 and non-significant (ns) at *p* > 0.05. To illustrate the distribution of some of the values, mean and median values were determined. As a measure for the dispersion of a random variable, the standard deviation around its mean was used. Mean values were compared using the non-parametric Mann-Whitney test.

## Outcomes

Outcomes were collected for 69 patients from the prospective electronic medical records (51/18 AAA/colorectal, respectively). Current CT images of the abdomen were available for 10/7 patients, respectively; these were used to objectively investigate the clinical diagnosis “no incisional hernia” or “incisional hernia”. A total of 30 patients were contacted via questionnaire. Mean follow-up in the aortic aneurysm group was 34.5 ± 18.1 months and 35.7 ± 21.4 months in the colorectal procedure group. No significant difference could be seen between the two groups in terms of mean follow-up in months.

Table 2 shows the age and gender distribution between the patient groups. There were significantly more females in the colorectal procedure group compared with the aortic aneurysm group.

**Table 2:** Sex and age distribution of the total cohort

Patient cohort (*n* = 99)	Total	AAA	Colo	*p*-value
*Sex (male/female)*	77/22	63/9	14/13	**<0.05**
*Age in years (M* *±* *SD)*	67.7 ± 8	67.8 ± 8.6	67.4 ± 5.9	n. s.
*Median age (years)*	68	68	67	–
*Preoperative hemoglobin (g/dl* *±* *SD)*	13.6 ± 2.2	14.11 ± 2.1	12.1 ± 1.9	**<0.05**
*Preoperative albumin (g/dl* *±* *SD)*	4 ± 0.6	4.2 ± 0.6	3.6 ± 0.7	**<0.05**
*ASA score* *±* *SD*	2.7 ± 0.7	2.8 ± 0.7	2.6 ± 0.7	n. s.
– Maximum value	5	5	4	–
– Minimum value	1	2	1	–
– Median	3	3	3	–
*Surgery time (min* *±* *SD)*	240.2 ± 135.1	266.6 ± 148	169.6 ± 42.5	**<0.05**
*ICU (days* *±* *SD)*	5.2 ± 15.9	4 ± 9.8	8.5 ± 26	**<0.05**
*Postoperative inpatient stay (days* *±* *SD)*	18.8 ± 16.9	17.3 ± 11.5	22.6 ± 26.5	n. s.

*AAA* abdominal aortic aneurysm, *ASA* American Society of Anaesthesiologists, *Colo* colorectal procedure, *ICU* intensive care unit, *M* mean, *n.s.* not significant, *SD* standard deviation

### Primary outcome: Incisional hernia development

Table 3 shows the incidence of incisional hernia in relation to the time of diagnosis and primary surgical procedure. The overall rate of incisional hernia was 17.2%: 4.0% of all patients developed a hernia within 180 days and 13.1% after 180 days. In all, 13.9% of the AAA group and 25.9% of the colorectal procedure group developed incisional hernia. There was no significant difference between the two patient groups in terms of the incidence of incisional hernia.

**Table 3:** The incidence of incisional hernia

Patient collective (*n* = 99)	Total		AAA		Colo		*p*-Value
	*n*	%	*n*	%	*n*	%	
Patients	99	100	72	–	27	–	–
Hernia in general	17	17.2	10	13.9	7	25.9	n. s.
Hernia before 180 days	4	4	3	4.2	1	3.7	n. s.
Hernia after 180 days	13	13.1	7	9.7	6	22.2	n. s.

*AAA* abdominal aortic aneurysm, *Colo* colorectal procedure, *n.s.* not significant

Analysis of the sex distribution in relation to the incidence rate of incisional hernia revealed no significant difference between male and female patients (Table 4).

**Table 4:** The incidence of incisional hernia (IH) in relation to diagnosis and sex

	No IH		IH		*p*-value	Relative risk	Confidence interval (0.95)
	*n*	%	*n*	%			
*Patients included*	–	–	17	100	–	–	–
Aortic aneurysm	62	75.6	10	58.8	n. s.	0.778	0.513–1.180
Colorectal	20	24.4	7	41.2	n. s.	1.688	0.852–3.346
Male	62	75.6	15	88.2	n. s.	0.482	0.124–1.873
Female	20	24.4	2	11.8	n. s.	1.167	0.943–1.444

*n.s.* not significant

No significant correlation was seen between risk factors and the occurrence of incisional hernia in aortic aneurysm patients (Table 5).

**Table 5:** The incidence of incisional hernia (IH) in aortic aneurysm (AAA) patients depending on preoperative risk factors

	AAA (total)		No IH		IH		*p*-Value	Relative risk	Confidence interval (0.95)
	*n*	%	*n*	%	*n*	%			
*Patients*	72	100	–	–	–	–	–	–	–
Male gender	63	87.5	53	85.5	10	100	n. s.	1.18	1.056–1.296
Age >50 years	72	100	62	100	10	100	n. s.	–	–
BMI >30 kg/m^2^	3^a^	4.3	3	5	0	0	n. s.	–	–
Nicotine use	34	47.2	30	48.4	4	40	n. s.	0.827	0.371–1.842
Diabetes mellitus	10	13.9	9	14.5	1	10	n. s.	0.689	0.098–4.866
Hypertension	57	79.2	48	77.4	9	90	n. s.	1.163	0.909–1.487
Renal failure	13	18.1	10	16.1	3	30	n. s.	1.86	0.617–5.609
Cortisone use	12	16.7	11	17.7	1	10	n. s.	0.564	0.081–3.903
Malignant (previous) disease	3	4.2	3	4.8	0	0	n. s.	–	–
Lung disease	17	23.6	14	22.6	3	30	n. s.	1.329	0.464–3.808
Collagen disease	–	–	–	–	–	–	–	–	–
Preoperative Hb <10 g/dl	3^b^	4.2	2	3.3	1	10	n. s.	3.05	0.304–30.587
Preoperative albumin <3.5 g/dl	4^c^	9.3	3	8.3	1	14.3	n. s.	1.714	0.207–4.188
History of hernia	7	9.7	7	11.3	0	0	n. s.	–	–
2nd Procedure/1 month	13	18.1	9	14.5	4	40	n. s.	2.756	1.044–7.270
Emergency surgery	10	13.9	8	12.9	2	20	n. s.	1.55	0.383–6.274
Previous abdominal surgery	16	22.2	15	24.2	1	10	n. s.	0.413	0.061–2.794

*BMI* body mass index, *Hb* hemoglobin, *n.s.* not significant

^a^*n* (valid) = 70

^b^*n* (valid) = 71

^c^*n* (valid) = 43

Table 6 presents the risk factors for the occurrence of incisional hernia following colorectal resection. Here again, there is no significant correlation between risk factors and the incidence of incisional hernia (Table 6).

**Table 6:** The incidence of incisional hernia (IH) in colorectal procedures depending on preoperative risk factors

	Colorectal (total)		No IH		IH		*p*-Value	Relative risk	Confidence interval (0.95)
	*n*	%	*n*	%	*n*	%			
*Patients*	27	100	–	–	–	–	–	–	–
Male gender	14	51.9	5	71.4	9	45	n. s.	0.63	0.321–1.236
Age >50 years	27	100	7	100	20	100	n. s.	–	–
BMI >30 kg/m^2^	1	3.7	0	0	1	5	n. s.	–	–
Nicotine use	4	14.8	1	14.3	3	15	n. s.	1.05	0.129–8.519
Diabetes mellitus	10	37	3	42.9	7	35	n. s.	0.817	0.288–2.318
Hypertension	18	66.7	2	28.6	16	80	< 0.05	2.8	0.850–9.219
Renal failure	6	22.2	2	28.6	4	20	n. s.	0.7	0.162–3.023
Cortisone use	5	18.5	1	14.3	4	20	n. s.	1.4	0.187–10.503
Malignant (previous) disease	2	7.4	1	14.3	1	5	n. s.	0.35	0.025–4.879
Lung disease	3	11.1	1	14.3	2	10	n. s.	0.7	0.074–6.581
Collagen disease	27	100	7	100	20	100	–	–	–
Preoperative Hb <10 g/dl	4^a^	15.4	2	28.6	2	10.5	n. s.	0.368	0.064–2.137
Preoperative albumin <3.5 g/dl	5^b^	33.3	2	66.7	3	25	n. s.	0.375	0.106–1.329
History of hernia	6	22.2	1	14.3	5	25	n. s.	1.75	0.245–12.510
2nd Procedure/1 month	10	37	3	42.9	7	35	n. s.	0.817	0.288–2.318
Emergency surgery	14	51.9	5	71.4	9	45	n. s.	0.63	0.321–1.236
Previous abdominal surgery	10	37	3	42.9	7	35	n. s.	0.817	0.288–2.318

*BMI* body mass index, *n.s.* not significant, *Hb* hemoglobin

^a^*n* (valid) = 26

^b^*n* (valid) = 15

### Secondary outcomes

**Fig. 2:**
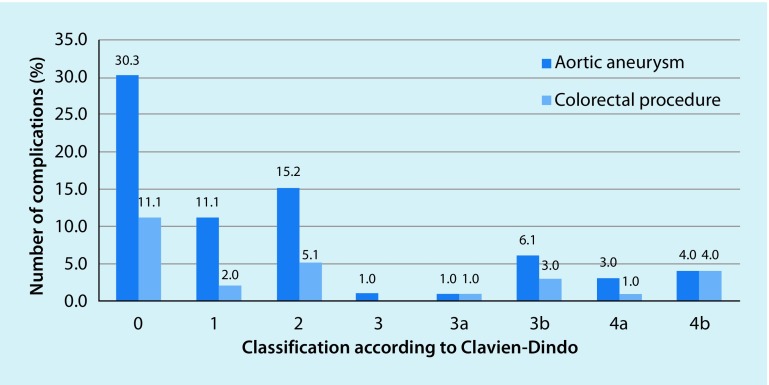
Overview of the distribution of postoperative complications according to Clavien and Dindo in relation to the diagnosis [12]. No significant differences were seen between the two groups. No postoperative classification could be determined for one patient. This is not shown for reasons of clarity

Postoperative complications are shown in Table 7. None of the postoperative complications in either group affected the development of incisional hernia. Fig. 2 shows the distribution of postoperative complications according to severity in line with the CDC [12]; these were also comparably distributed in both cohorts. No significant differences were seen in terms of the incidence of incisional hernia and postoperative non-intensive medical complications. The relative risk of developing an incisional hernia in the case of complications requiring revision was increased by 34%, without, however, reaching significance in this collective.

**Table 7:** The incidence of postoperative complications in the aortic aneurysm (AAA) and colorectal groups

	AAA		Colorectal		*p*-Value
	*n*	%	*n*	%	
*Complications/patients*	*72*	*100*	*27*	*100*	*–*
No complications	30	41.7	11	40.7	n. s.
Wound infection	11	15.3	9	33.3	n. s.
Seroma	2	2.8	0	0	n. s.
Secondary wound healing	3	4.2	5	18.5	n. s.
Oral anticoagulation	65	90.3	12	44.4	n. s.
Other complications	35	48.6	13	48.1	n. s.
Complications requiring revision	7	9.7	8	29.6	n. s.

*n.s.* not significant

## Discussion

### Key messages of the study

The matched-control comparison revealed no differences between the two groups in relation to the primary endpoint, i. e., the development of incisional hernia. The incidence of incisional hernia following aortic aneurysm surgery tended to be even lower compared with the colorectal group. According to Clavien-Dindo score, complications in both groups were comparable. In neither group was it possible to establish and influence of the postoperative course and postoperative complications on the development of incisional hernia. These results confirm earlier results obtained by Israelsson [11], who found that patients do not have a greater risk for incisional hernia following aortic aneurysm repair than patients following colorectal procedures with accesses of similar dimensions.

#### Evaluation and critical review of own results

The surgical technique of abdominal wall closure is of crucial importance for the postoperative incidence of incisional hernia. In this study, an incidence rate of 13.9% was seen in the aortic aneurysm group. Incidence rates of between 6% [11] and 35% in aortic aneurysm patients are reported in the literature [11, 13, 14]. The not significantly higher rate of incisional hernia in the colorectal group, as well as the longer stay in the intensive care unit, can probably be accounted for by underlying diseases, such as sigmoid diverticulitis in this cohort. The most important weaknesses of this study are its retrospective data collection and the low patient number; however, it will not become easier to include patients undergoing open aortic aneurysm repair in studies in the future, given that endovascular techniques have become standard in the last 10 years. For this reason, the present study is of value despite its low patient number. What is of particular relevance about this study is that, for the first time, incisional hernia incidence was investigated while taking into account the anatomy of access, i. e., not only medial morphology, but also the xiphopubic extension of the incision was an inclusion criterion. These criteria can be found in the Würzburg incisional hernia classification [7] and the European Hernia Society (EHS) classification [9]. All cases of shorter medial access were eliminated in the control group, resulting in a highly homogeneous and comparable study population.

#### Explanatory models for the development of incisional hernia and aortic aneurysm

Collagen I and III play a particularly important role in scar formation: collagen III is metabolized primarily in the early phase of scar formation, collagen I in the late phase [15]. Collagen I is essential for scar stability. It is known that hernia patients have a lower collagen I:III ratio in favor of predominantly high collagen III [4, 5, 16]. An increased incidence of incisional hernia has also been observed in hereditary diseases involving collagen defects [17–20]. Matrix metalloproteases (MMP) play a crucial role in the alteration of collagens. They are particularly important in the regulation of growth and apoptotic processes [21, 22]. There appears to be significant overexpression of MMP-2 in incisional hernia [23]. Since collagen synthesis can be suppressed by inflammatory processes, such as postoperative sepsis, the functionality of the various MMP and collagens may be impaired by the wound milieu [24]. Current models of the development of aortic aneurysm work on the premise that the pathogenesis of aortic aneurysms is not based on a collagen defect as previously assumed [6]: an elastase defect has been postulated [8], while more recent experimental studies also focused on inflammation as the cause of aortic wall weakening during the process of aneurysm development [25]. Ultimately, the molecular etiology of aneurysm development remains unclear; however, the collagen weakness assumed in the past to be the cause of AAA is highly unlikely according to current knowledge.

#### Taking a careful approach to abdominal wall closure

One possible factor that affects the low incidence of incisional hernia following aortic surgery in the authors’ department is related to the fact that the vascular surgeons there have broad general surgical training. The importance of meticulous abdominal wall closure cannot be disregarded in this context. Evidence shows that maintaining a suture length–wound length ratio of 4:1 [26, 27], using a continuous suture with slowly absorbable or non-absorbable suture material [26, 28–37], maintaining a distance from needle puncture to wound edge of at least 1 cm [30], as well as distances of less than 1 cm between two punctures [31], can reduce the incidence of incisional hernia. Ever since the publication of works by Israelsson in the mid 1990s, it has been the accepted wisdom that less can be more: by using smaller needles and small tissue bites, incisional hernia incidence in medial laparotomy following aortic aneurysm repair can be reduced from 21% to 10% (*p* < 0.01), and to as little as 5.2% in selected patients [11, 31–33]. Although a recent multi-center randomized study was not able to achieve quite these results, it confirmed the superiority of the small-bites technique (13%) compared with the large-bites technique (21%) at 1 year (*p* = 0.022; OR 0.52; CI 95% 0.31–0.87; *p* = 0.0131) [34].

### Is prophylactic mesh placement beneficial in AAA?

The surgical management of hernias has moved away from the conventional suture technique in recent years in favor of repair using mesh [35]. According to these positive results, it has been postulated that maybe prophylactic mesh placement could significantly reduce the incidence of incisional hernias in high-risk patients, for example, in obese patients. A recent meta-analysis of prophylactic mesh placement in the setting of open bariatric procedures also revealed a positive effect for mesh when the available cohort studies were included (OR 0.39; CI 95% 0.13–0.68; *p* = 0.004); however, if one considers only the four randomized controlled trials (RCT) included in the meta-analysis, prophylactic mesh placement conferred no benefit (OR 0.52; CI 95% 0.24–1.16; *p* = 0.104) [36]. That particular meta-analysis did not include the recently published RCT conducted by Muysoms et al. Here, the incidence of incisional hernia at 2 years (with approximately 10% loss to follow-up) in the control group (continuous suture using the large-bites technique) was 28% and in the prophylactic mesh group (retromuscular mesh placement) 0% (*p* < 0.001); however, retromuscular mesh placement is considerably more complex and the advantages of suture techniques are not exhausted with the large-bites control group [37]. This study needs to be re-interpreted and re-weighted in the light of the small-stitches technique.

In summary, it must be concluded that according to current evidence, the meticulousness of abdominal wall closure based on Israelsson’s recommendations [11, 32] appears to be the most appropriate approach irrespective of the surgical indications.

## Conclusion

- After abdominal operations the development of an undesired and, depending on the findings, a high-risk incisional hernia occurs in 2–15% of cases. Abdominal aortic aneurysms (AAA) have an inflammatory origin. According to the current state of knowledge collagen metabolism can be excluded as a trigger factor.
- Patients with AAA do not have an increased risk of incisional hernia in comparison to patients after a benign colorectal intervention. The incidence rates with respect to age, sex or risk factors do not show significant differences between both groups.
- The surgical technique for abdominal wall closure is of decisive importance for the postoperative incidence of incisional hernias. Postoperative complications had no influence on their formation.
  There is a trend in the literature, suggesting that prophylactic implantation of a mesh may reduce the incidence of incisional hernias in patients at risk.
- The retrospective data collation and the low number of patients are weak points of this study; however, in the future it will not be easier to include patients with open treatment of aortic aneurysms in studies because now endovascular procedures have become the standard.
